# Severe Autoinflammatory Manifestations and Antibody Deficiency Due to Novel Hypermorphic *PLCG2* Mutations

**DOI:** 10.1007/s10875-020-00794-7

**Published:** 2020-07-15

**Authors:** Andrea Martín-Nalda, Claudia Fortuny, Lourdes Rey, Tom D. Bunney, Laia Alsina, Ana Esteve-Solé, Daniel Bull, Maria Carmen Anton, María Basagaña, Ferran Casals, Angela Deyá, Marina García-Prat, Ramon Gimeno, Manel Juan, Helios Martinez-Banaclocha, Juan J Martinez-Garcia, Anna Mensa-Vilaró, Raquel Rabionet, Nieves Martin-Begue, Francesc Rudilla, Jordi Yagüe, Xavier Estivill, Vicente García-Patos, Ramon M. Pujol, Pere Soler-Palacín, Matilda Katan, Pablo Pelegrín, Roger Colobran, Asun Vicente, Juan I. Arostegui

**Affiliations:** 1 Pediatric Infectious Diseases and Immunodeficiencies Unit, Vall d’Hebron Institut de Recerca, Hospital Universitari Vall d’Hebron, Barcelona, Spain; 2 Jeffrey Modell Diagnostic and Research Center for Primary Immunodeficiencies, Barcelona, Spain; 3 Department of Pediatrics, Hospital Sant Joan de Deu, Esplugues, Spain; 4 Institut de Recerca Hospital Sant Joan de Déu, Universitat de Barcelona, Esplugues, Spain; 5 Department of Pediatrics, Hospital Alvaro Cunqueiro, Vigo, Spain; 6 Institute of Structural and Molecular Biology, University College London, London, UK; 7 Department of Allergy and Clinical Immunology Clinical Immunology and Primary, Immunodeficiencies Unit, Hospital Sant Joan de Déu, Esplugues, Spain; 8 Clinical Immunology Unit, Hospital Sant Joan de Déu-Hospital Clínic, Barcelona, Spain; 9 ARUK Drug Discovery Institute, University College London, London, UK; 10 Department of Immunology-CDB (esc 4-pl 0), Hospital Clínic, Villarroel, 170, 08036 Barcelona, Spain; 11 Allergy Section, Hospital Universitari Germans Trias i Pujol, Autonomous University of Barcelona, Badalona, Spain; 12 Genomics Core Facility, Experimental and Health Sciences Department, Universitat Pompeu Fabra, Barcelona, Spain; 13 Department of Immunology, Hospital del Mar, Institut Mar d’Investigacions Mèdiques, Barcelona, Spain; 14 Institut d’Investigacions Biomèdiques August Pi i Sunyer, Barcelona, Spain; 15 School of Medicine, Universitat de Barcelona, Barcelona, Spain; 16 Instituto Murciano de Investigación Biosanitaria IMIB-Arrixaca, Hospital Clínico Universitario Virgen de la Arrixaca, Murcia, Spain; 17 Department of Genetics, Microbiology and Statistics, Faculty of Biology, University of Barcelona, IBUB, IRJSD, CIBERER, Barcelona, Spain; 18 Department of Pediatric Ophthalmology, Hospital Universitari Vall d’Hebron, Vall d’Hebron Institut de Recerca, Barcelona, Spain; 19 Histocompatibility and Immunogenetics Laboratory, Blood and Tissue Bank, Barcelona, Spain; 20 Transfusional Medicine Group, Vall d’Hebron Research Institute, Autonomous University of Barcelona, Barcelona, Spain; 21 Quantitative Genomic Medicine Laboratories (qGenomics), Esplugues del Llobregat, Barcelona, Catalonia Spain; 22 Department of Pediatric Dermatology, Hospital Universitari Vall d’Hebron, Vall d’Hebron Institut de Recerca, Barcelona, Spain; 23 Department of Dermatology, Hospital del Mar, Institut Mar d’Investigacions Mèdiques, Universitat Autonoma de Barcelona, Barcelona, Spain; 24 Universitat Autonoma de Barcelona, Barcelona, Spain; 25 Immunology Division, Department of Clinical and Molecular Genetics, Hospital Universitari Vall d’Hebron, Vall d’Hebron Research Institute, Barcelona, Spain; 26 Department of Cell Biology, Physiology and Immunology, Autonomous University of Barcelona, Barcelona, Spain; 27 Department of Pediatric Dermatology, Hospital Sant Joan de Deu, Esplugues, Spain

**Keywords:** Autoinflammatory diseases, APLAID, PLCγ2, inflammasome, caspase-1, interleukin-1, agammaglobulinemia

## Abstract

Autoinflammatory diseases (AIDs) were first described as clinical disorders characterized by recurrent episodes of seemingly unprovoked sterile inflammation. In the past few years, the identification of novel AIDs expanded their phenotypes toward more complex clinical pictures associating vasculopathy, autoimmunity, or immunodeficiency. Herein, we describe two unrelated patients suffering since the neonatal period from a complex disease mainly characterized by severe sterile inflammation, recurrent bacterial infections, and marked humoral immunodeficiency. Whole-exome sequencing detected a novel, de novo heterozygous *PLCG2* variant in each patient (p.Ala708Pro and p.Leu845_Leu848del). A clear enhanced PLCγ2 activity for both variants was demonstrated by both ex vivo calcium responses of the patient’s B cells to IgM stimulation and in vitro assessment of PLC activity. These data supported the autoinflammation and PLCγ2-associated antibody deficiency and immune dysregulation (APLAID) diagnosis in both patients. Immunological evaluation revealed a severe decrease of immunoglobulins and B cells, especially class-switched memory B cells, with normal T and NK cell counts. Analysis of bone marrow of one patient revealed a reduced immature B cell fraction compared with controls. Additional investigations showed that both *PLCG2* variants activate the NLRP3-inflammasome through the alternative pathway instead of the canonical pathway. Collectively, the evidences here shown expand APLAID diversity toward more severe phenotypes than previously reported including dominantly inherited agammaglobulinemia, add novel data about its genetic basis, and implicate the alternative NLRP3-inflammasome activation pathway in the basis of sterile inflammation.

**Electronic supplementary material:**

The online version of this article (10.1007/s10875-020-00794-7) contains supplementary material, which is available to authorized users.

## Introduction

Autoinflammatory diseases (AIDs) constitute a specific group of primary immunodeficiency diseases (PID) characterized by recurrent episodes of systemic sterile inflammation mainly mediated by cells of innate immunity [1]. At present, around 30 different monogenic AIDs have been molecularly elucidated, with some of the novel ones also displaying features of dysregulated B cell response either as circulating autoantibodies or as reduced antibody production [2–5]. The extremely rare, dominantly inherited PLCγ2-associated antibody deficiency and immune dysregulation (PLAID) and autoinflammation and PLCγ2-associated antibody deficiency and immune dysregulation (APLAID) may be included among those rare monogenic AIDs combining sterile inflammation and humoral immunodeficiency. Both diseases are linked to hypermorphic, pathogenic variants in the *PLCG2* gene encoding the key signal transduction enzyme PLCγ2. They are clinically characterized by early-onset skin inflammation and recurrent infections. In addition, patients with APLAID often develop ocular and lung inflammation, enterocolitis, and interstitial pneumonitis but unlike PLAID, they do not present with cold urticaria or autoimmunity [6–9]. However, because only a few APLAID cases have been characterized so far, it is not clear to what extent their clinical manifestations could differ or overlap with other immune disorders. Similarly, the range of genetic changes (in *PLCG2* or in genes regulating associated signal transduction pathways) and their impact on immune cell functions have not been defined.

In the present study we describe two unrelated patients with early-onset severe skin and eye inflammation, and recurrent bacterial infections secondary to antibody deficiency. Further genetic and functional studies are consistent with APLAID and consolidate and expand the key features and underpinning molecular mechanisms for this diagnosis.

## Patients and Methods

The ethics committees of Hospital Sant Joan de Déu, Hospital Universitari Vall d’Hebron and Hospital Clínic, all in Barcelona (Spain), approved the study. Written informed consent for participation in the study was obtained from patients’ parents. Blood samples from patients and unaffected relatives were collected for molecular studies, which were performed in accordance with the Declaration of Helsinki.

### Flow Cytometry Studies

Peripheral blood mononuclear cells (PBMCs) were isolated by Ficoll gradient (Fresenius-Kabi Norge, Norway) and stained for cell surface markers using fluorochrome-conjugated antibodies (Supplementary Table S1). Samples were acquired using a FACSCanto II cytometer (BD Biosciences, USA), and data were analyzed with CellQuest software (BD Biosciences, USA).

### Genetic Analyses

DNA samples were extracted from peripheral blood using a QIAmp DNA Blood Mini Kit (Qiagen, Germany). Libraries were prepared using TruSight One kit (Illumina, USA) in Family 1 and SureSelect Human All exon V2 kit (Agilent Technologies, USA) in Family 2 following manufacturer’s instructions. Paired-end sequencing was performed on an Illumina Genome Analyzer II platform (Illumina, USA). Reads were mapped against the human reference genome hg19 using the BWA software and analyzed with the GATK Unified Genotyper v2.8. Amplicon-based deep sequencing of specific exons of the *PLCG2* gene (RefSeq NM_002661.3) was performed as previously described to evaluate parental gene mosaicism [10]. For Sanger sequencing, specific exons of the *PLCG2* gene were amplified by in house–designed PCR (primers listed in Supplementary Table S2), purified with Illustra ExoStar 1-Step kit (GE Healthcare, USA), bidirectional sequenced using ABI BigDye® Terminator v3.1 Cycle Sequencing Kit (Applied Biosystems, USA) and run on an automated ABI 3730XL analyzer (Applied Biosystems, USA).

### Analyses of Ca^2+^ Flux in B Cells and Measurements of Phosphoslipase-C Activity

Intracellular Ca^2+^ flux was measured by flow cytometry after labeling with FLUO-3 AM (Invitrogen, USA) as previously described with slight modifications [6].

For the measurements of phosphoslipase-C (PLC) activity, COS-7 cells were cultured in DMEM (Sigma-Aldrich, USA) containing 10% (v/v) FBS and 2.5 mM glutamine (growth media). Cells were grown as a monolayer at 37 °C in 5% CO_2_. COS-7 cells were seeded into 96-well plates at a density of 7500 cells/well in 0.1 mL of growth media and incubated overnight. Fresh media was applied and the cells transfected with plasmid DNA at 100 ng/well that had been diluted in 5 μL jetPRIME® buffer and 0.2 μL jetPRIME® (Polyplus Transfection, France) that was prepared as instructed by the manufacturer. The DNA concentration was kept constant by adding empty plasmid. For the PLCγ2 expression plasmids, the full-length ORF of human *PLCG2* was cloned into the vector pTriEx4 using Gateway technology (Thermo Fisher). Mutations and deletions were prepared using the site-directed mutagenesis kit (Agilent) following manufacturer’s instructions. The ORFs of all constructs were fully sequenced prior to a Maxiprep being performed to generate the plasmid DNA for transfection. Each PLCγ2 construct was transfected at 4 concentrations in triplicate as outlined in the figures. Twenty-four hours post-transfection, the media was removed and replaced with growth media without FBS but containing 0.25% (w/v) fatty acid free BSA. The COS-7 cells were then incubated for further 24 h. Subsequently, the media was replaced with growth media without FBS but containing 50 mM LiCl with and without 100 ng/mL EGF and incubated for further 1 h. The media was aspirated and replaced by 25 μL of stimulation buffer (20 mM HEPES.OH, 2 mM CaCl_2_, 1 mM MgCl_2_, 8.4 mM KCl, 292 mM NaCl, 11 mM glucose and 100 mM LiCl, pH 7.4) followed by 25 μL of lysis buffer (50 mM HEPES.OH, 0.8 M KF, 0.2% (w/v) BSA and 1% (v/v) Triton-X-100, pH 7.0). The cells were lysed for 30 min at room temperature on an orbital shaker. Seven microliters of the cell lysate was pipetted in duplicate into a white 384-well plate (Greiner Bio-One GmbH, Austria) followed by 1.6 μL of IP1-d2 (Cisbio, France). After 5 min, 1.6 μL of anti-IP1-Cryptate (Cisbio, France) was added and the plate sealed and incubated at room temperature for 1 h. The plate was read on a PHERAstar (BMG Labtech, Germany) plate reader in HTRF mode, and the data converted to IP_1_ concentration using a standard curve generated following manufacturer’s instructions. Data for the measured PLC activity represent the standard error of the mean of transfections performed in triplicate.

Quantities of expressed proteins were measured using a WES Western Blotting system (Protein Simple, USA). For this, a further 96-well plate was transfected identically to the plate used in the IP_1_ assay described above. Forty-eight hours post-transfection, the cells were washed once in ice-cold PBS and subsequently lysed in 20 μL RIPA buffer (Thermofisher, USA) containing a protease and phosphatase inhibitor cocktail (Thermofisher, USA). The cells were freeze-thawed at − 20 °C and subsequently 4 μL of each well loaded on a WES Western Blotting. Proteins were detected with a 1:150 dilution of the anti-PLCγ2 antibody sc5283 (Santa Cruz) and a 1:150 dilution of the anti-β-actin antibody 13E5 (Cell Signaling Technology). For the comparison of PLC activity of different PLCγ2 variants, the expression levels were quantitated and points with the same protein expression used, as previously described [11]. The differences were confirmed using the 2 tailed *t* test.

### Detection of Intracellular ASC Specking and Active Caspase-1

PBMCs were treated with either nothing or *E. coli* LPS serotype 055:B5 (Sigma-Aldrich, USA; 1 μg/mL, 2 h at 37 °C). LPS-primed PBMCs were then stimulated with either nothing, ATP (3 mM), or nigericin (10 μM) for 30 min at 37 °C. Stimulated PBMCs were fixed with 2% paraformaldehyde (Sigma-Aldrich, USA) and stained for the detection of intracellular ASC specks by TOFIE as previously described [12], using the rabbit polyclonal antibody anti-ASC (N-15)-R (Santa Cruz Biotechnology, USA).

For active caspase-1 detection, PBMCs were incubated for 1 h with FLICA660 reagent (ImmunoChemistry Technologies, USA) and fixed following manufacturer recommendations. Monocytes were detected with the APC-vio770-conjugated anti-human CD33 antibody (Miltenyi Biotec, Germany) and with the APC-Cy7-conjugated anti-human CD14 antibody (TONBO Biosciences, USA). Stained cells were acquired on a FACSCanto cytometer (BD Biosciences, USA). Cytokines were measured in cell supernatants using a custom bead-based multiplex Luminex immunoassay (eBioscience, USA). Heat maps representing cytokine expression profiles were created using Morpheus software (Broad Institute, Cambridge, USA).

## Results

### Clinical Description

#### Family 1

**Fig. 1:**
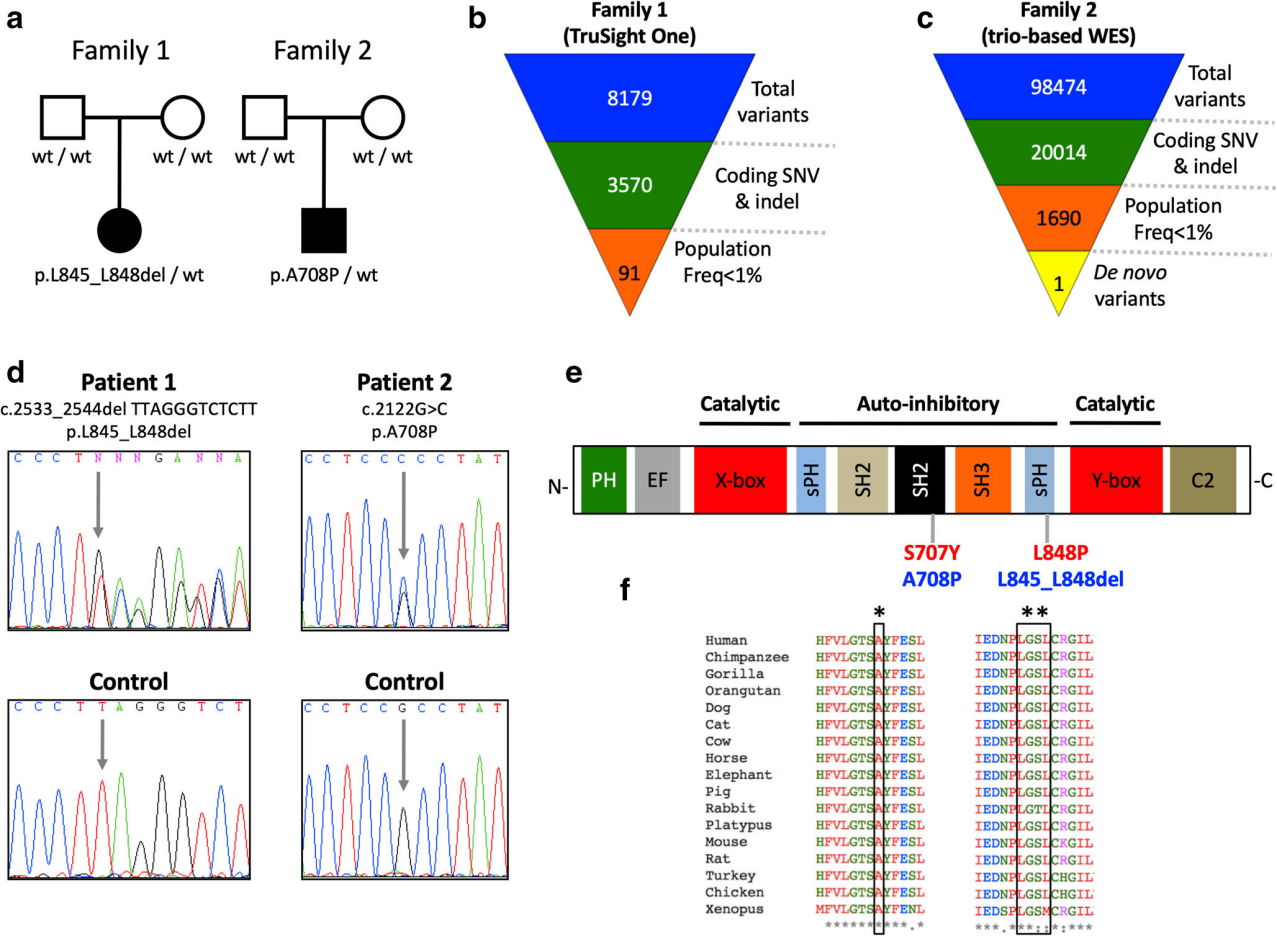
Familial pedigrees and features of *PLCG2* variants. **Panel a** Pedigrees of enrolled families. Black filled symbols represent affected individuals, open symbols unaffected individuals, squares male individuals and circles female individuals. **Panels b**, **c** Schemes of filtering of the next-generation sequencing strategies used in the enrolled families. **Panel d** Sanger sense chromatograms of the *PLCG2* gene from patients (upper boxes) and from wild-type healthy subjects (bottom boxes). Gray arrows indicate the *PLCG2* variants detected in the patients. **Panel e** Scheme of structural domains of phospholipase Cγ2 protein. The already known APLAID-associated *PLCG2* mutations are showed in red fonts, while the *PLCG2* variants described in the present work are displayed in blue fonts. **Panel f** Multiple sequence alignment of human phospholipase Cγ2 and sixteen orthologues. The single asterisk represents the amino acid residue 708 of human phospholipase Cγ2, while two asterisks indicate the amino acid residues 845–848

Patient 1 has been first described in 2007 [13] and is now a 16-year-old girl. She was born from healthy parents at 40 weeks of gestation by cesarean section due to loss of fetal wellbeing (pedigree in Fig. 1a). The main features of her disease included early-onset severe cutaneous and eye inflammation and recurrent respiratory infections (Table 1).

**Table 1:** Summary of clinical and immunological features of enrolled patients and comparison with reported APLAID patients (Zhou et al. [7]; Neves et al. [8]; Morán-Villaseñor et al. [9]

	Present study		Zhou et al.		Neves et al.	Moran-Villaseñor et al.
	Patient 1	Patient 2	Patient II-1	Patient III-2	Patient 1	Patient 1
Clinical features						
Age at disease onset	Birth	5th day of life	Infancy	Infancy	1st week of life	3rd day of life
Cutaneous lesions	Erythematous plaques, vesiculopustular and ulcerative lesions, ulcerative granulomata, hyperpigmentation *cutis laxa*	Maculo-papular eruption, erythematous plaques, urticarial-like lesions, vesiculo-pustular lesions, hyperpigmentation *cutis laxa*	Epidermolysis bullosa–like eruption, erythematous plaques, vesiculopustular lesions	Epidermolysis bullosa–like eruption, erythematous plaques, vesiculopustular lesions	Vesiculopustular rash, cutaneous granulomas, blistering skin rash *cutis laxa*	Erythematous pustules, yellow-pink papules, pseudovesicles, acral hemorrhagic blisters, cutaneous granulomas
Eye inflammation	Bilateral corneal erosions, corneal limbitis, corneal nodules, haze bilateral conjunctivitis, bilateral episcleritis	Bilateral episcleritis, corneal limbitis, bilateral episcleritis	-	Corneal small blisters, corneal erosions, corneal ulcerations, intraocular hypertension cataracts	Recurrent eye inflammation, posterior uveitis, ocular hypertension	Non-purulent conjunctival erythema
Lung involvement	Bronchiectasis, recurrent episodes of hemoptysis	-	Interstitial pneumonitis	Interstitial pneumonitis	Interstitial pneumonitis	-
Joint involvement	-	-	Arthralgias	Arthralgias	-	Arthralgias
Gastrointestinal involvement	-	-	Enterocolitis	Recurrent abdominal pain, bloody diarrhea, ulcerative colitis	Bloody diarrhea, early-onset inflammatory bowel disease	Recurrent episodes of diarrhea
Infections	Recurrent sinopulmonary infections, herpetic stomatitis, recurrent bacterial and fungal skin infections	Recurrent bronchitis, recurrent suppurative otitis, pneumonias, perineal dermatitis, acute gastroenteritis (*C. jejuni)*	Recurrent sinopulmonary infections, cellulitis	Cellulitis	Recurrent infections (pneumonia, ear, sinus)	Recurrent upper respiratory infections
Immunodeficiency	Yes	Yes	Yes	Yes	Yes	Yes
Immunological features						
T cells	Normal	Normal	Normal	Normal	Normal	Normal
B cells	Very low/absent	Very low	Normal	Low	Low	Low
NK cells	Normal	Normal	Normal	Normal	Normal	Low
IgG	Very low	Normal	Normal	Normal	Low	Low
IgA	Very low	Normal	Low	Low	Low	Low
IgM	Very low	Low-Very low	Low	Low	Low	Low
IgE	Very low	Low	n.a.	n.a.	n.a.	n.a.
Autoantibodies	Negative	Negative	Negative	Negative	Negative	Negative
Genetic features						
*PLCG2* genotype	p.L845_L848del/wt	p.A708P/wt	p.S707Y/wt	p.S707Y/wt	p.L848P/wt	p.L848P/wt

**Fig. 2:**
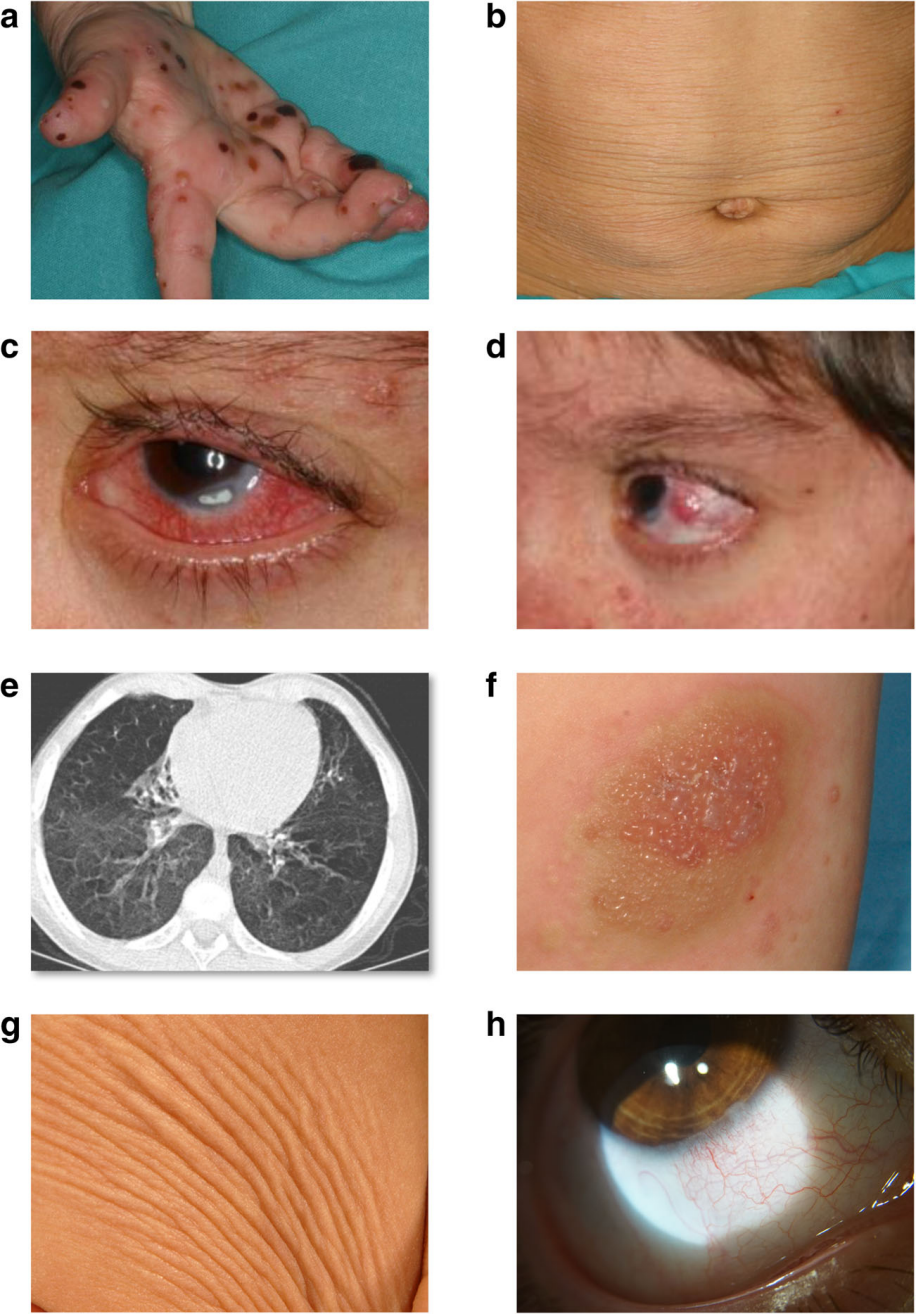
Description of cutaneous, pulmonary, and ocular inflammatory manifestations in patients. **Panel a** Multiple papules and serosal and hemorrhagic vesicles on the hands and palms detected in patient 1 at the age of 4 years. **Panel b** Large areas of *cutis laxa* in the abdominal region detected in patient 1 at the age of 7 years. **Panels c, d** Ocular inflammatory lesions including intense bilateral conjunctivitis, keratitis, episcleritis, and nodules in the sclera detected over the course of the disease in patient 1. **Panel e** Bronchiectasis detected in patient 1. **Panel f** Blistering inflammatory cutaneous lesions in the leg detected in patient 2 at the age of 6 months. **Panel g** Areas of *cutis laxa* detected in patient 2 at the age of 6 years. **Panel h** Ocular inflammatory lesions including conjunctivitis and corneal limbitis detected in patient 2 at the age of 7 years

Skin lesions appeared as early as the first day of life as numerous papulo-vesicular lesions, which became generalized during the following days requiring admission into pediatric intensive care unit. These lesions have been nearly continuously present, with exacerbations, occasionally hemorrhagic and complicated with infections, ulcerated lesions, and ulcerative granulomata (Fig. 2a). In recent years, large areas of *cutis laxa* and hyperpigmentation were detected (Fig. 2b). At 2.5 years of age, bilateral conjunctivitis, corneal erosions, and nodules appeared (Fig. 2c, d).

The absence of circulating immunoglobulins was detected during the first year of life (Table 2). Intravenous immunoglobulin (IVIG) replacement therapy (IVIGs; 400 mg/kg q3w) was then started and has been maintained until present. Despite this treatment, multiple infections were detected (cutaneous infections, acute gastroenteritis, periodontitis, herpetic stomatitis, bronchitis, and pneumonia). At 4 years of age, multiple central bronchiectases were detected on a CT scan (Fig. 2e), which subsequently progressed and provoked recurrent episodes of acute hemoptysis that required urgent embolization. As consequence of these lesions, the medium right lung lobe was surgically excised at the age of 13 years.

**Table 2:** Summary of immunological investigations performed in enrolled patients. Figures in brackets indicate the normal range of each parameter adjusted per age. Italic fonts indicate values lower than the normal range

	Patient 1					Patient 2				
Age at measurement	9 m	3 y 8 m	4y 3 m	5 y 11 m	11 y 6 m	5 m	1 y 1 m	3 y 7 m	4 y 7 m	5 y 6 m
Lymphocyte subpopulations										
Total lymphocytes (10^3^/μL)	n.a.	3.46 (2.0–8.0)	2.9 (2.0–8.0)	2.9 (2.0–8.0)	2.1 (1.2–5.2)	6.6 (4.0–13.5)	4.1 (4.0–10.5)	3.1 (2.0–8.0)	2.2 (2.0–8.0)	3.1 (2.0–8.0)
CD3^+^ (cels/μL)	n.a.	3044 (1400–3700)	2639 (1400–3700)	2726 (1400–3700)	1806 (1200–2600)	5742 (2500–5600)	3649 (2100–6200)	2666 (1400–3700)	1936 (1400–3700)	2759 (1400–3700)
CD3^+^ CD4^+^ (cels/μL)	n.a.	2145 (700–2200)	1827 (700–2200)	1783 (700–2200)	1197 (650–1500)	4290 (1800–4000)	2706 (1300–4300)	1364 (700–2200)	1056 (700–2200)	1364 (700–2200)
CD3^+^ CD8^+^ (cels/μL)	n.a.	1038 (490–1300)	754 (490–1300)	899 (490–1300)	546 (370–1100)	1452 (590–1600)	902 (620–2000)	1209 (490–1300)	792 (490–1300)	1209 (490–1300)
CD19^+^ (cels/μL)	n.a.	*35* (390–1400)	*17* (390–1400)	*6* (390–1400)	*13* (270–860)	*330* (430–3000)	*246* (720–2600)	*62* (390–1400)	*44* (390–1400)	*31* (390–1400)
CD16/56^+^ (cels/μL)	n.d.	138 (130–720)	208 (130–720)	130 (130–720)	182 (100–480)	462 (170–830)	*164* (180–920)	310 (130–720)	176 (130–720)	217 (130–720)
Immunoglobulin plasma levels										
IgG (mg/dL)	*125* (217–904)	439* (441–1135)	n.d.	871* (463–1236)	638* (639–1349)	173 (172–814)	537 (345–1213)	732 (441–1135)	696 (463–1236)	819 (463–1236)
IgA (mg/dL)	11 (11–90)	4.9* (22–159)	n.d.	< 10* (25–154)	< 10* (70–312)	21 (8–68)	58.8 (14–106)	139 (22–159)	83 (25–154)	102 (25–154)
IgM (mg/dL)	*4* (34–126)	8* (47–200)	n.d.	< 10* (43–196)	< 10* (56–352)	*8.2* (33–109)	82.9 (43–173)	*26* (47–200)	*21* (43–196)	*31.5* (43–196)
IgE (kU/L)	n.d.	< 10.0 (0.0–7.3)	< 10.0 (0.0–7.3)	< 10.0 (0.0–7.3)	n.d.	< 10.0 (0–140)	n.d.	< 10.0 (0–140)	n.d.	< 10.0 (0–140)
IgG subclass plasma levels										
IgG_1_ (mg/dL)	n.d.	n.v.*	n.v.*	n.v.*	n.v.*	*158* (170–950)	n.d.	n.d.	n.d.	n.d.
IgG_2_ (mg/dL)	n.d.	n.v.*	n.v.*	n.v.*	n.v.*	*19* (21–440)	n.d.	n.d.	n.d.	n.d.
IgG_3_ (mg/dL)	n.d.	n.v.*	n.v.*	n.v.*	n.v.*	25.6 (12.7–55.5)	n.d.	n.d.	n.d.	n.d.
IgG_4_ (mg/dL)	n.d.	n.v.*	n.v.*	n.v.*	n.v.*	*4* (5–16)	n.d.	n.d.	n.d.	n.d.
Post-vaccine antibodies										
Pneumovax-23	n.d.	n.v.*	n.v.*	n.v.*	n.v.*	n.d.	n.d.	n.d.	n.d.	Negative
Diphtheria	n.d.	n.v.*	n.v.*	n.v.*	n.v.*	n.d.	n.d.	n.d.	n.d.	Negative
Tetanus	n.d.	n.v.*	n.v.*	n.v.*	n.v.*	n.d.	n.d.	n.d.	n.d.	Negative
Autoantibodies	n.d.	Negative [1]	Negative [2]	n.d.	Negative [3]	n.d.	Negative [4]	Negative [5]	Negative [6]	n.d.

*Values obtained during intravenous immunoglobulin therapy [1]N.egative results for anti-transglutaminase (IgG and IgA) and antiendomisio (IgA) antibodies [2]N.egative results for antinuclear antibodies (ANA), anti-Ro, anti-La, anti-RNP, anti-Sm, anti-smooth muscle, anti-LKM, and anti-mitochondrial antibodies [3]N.egative results for antinuclear antibodies (ANA), anti-DNA, anti-Ro, anti-La, anti-RNP, anti-Sm, anti-Scl70, and anti-JO-1 antibodies [4]N.egative results for anti-transglutaminase antibodies [5]N.egative results for antinuclear antibodies (ANA), anti-DNA autoantibodies, and anti-neutrophil cytoplasmatic autoantibodies [6]N.egative results for anti-neutrophil cytoplasmatic autoantibodies. *m*, months; *y*, years; *n.a.*, not available; *n.d.*, not done; *n.v.*, not valuable

The patient received multiple treatments including antibiotics, retinoids, corticosteroids, etanercept (25 mg q1w for 5 years), and anakinra (100 mg q1d for 1.5 years). With the use of etanercept and anakinra, a partial control of skin inflammation was detected, with no improvement of ocular manifestations or immune defects. By contrast, a marked decrease of plasma C-reactive protein (CRP) was detected with etanercept (mean 6.86 mg/L; range 0.08–21-8) and anakinra (mean 0.10 mg/L; range 0.05–0.16) compared with periods in which these treatments were not administered (mean 13.32 mg/L; range 1.3–31.63).

#### Family 2

Patient 2 is a 9-year-old boy born from healthy parents (pedigree in Fig. 1a). Skin manifestations started during the first week of life as multiple erythematous macules, papules, and large plaques, mainly located at arms, abdomen, and thighs (Table 1). These lesions recurred with no periodicity, sometimes presenting as urticaria-like, vesicular or pustular lesions (Fig. 2f) or ulcerated or exudative plaques. Infections, minor traumas, vaccinations, and heat were identified as triggering or worsening factors. They were successfully treated with oral or topical corticosteroids (1 mg/kg q1d), and partially with dapsone (1 mg/kg q1d), and they healed leaving focal, wrinkled-appearing patches of *cutis laxa* and residual hyperpigmentation (Fig. 2g). Ocular inflammatory manifestations have recently appeared as bilateral red eye, corneal limbitis, and episcleritis (Fig. 2h).

Infections started at the age of 2 months as recurrent, mild viral bronchitis. Since the age of 4 years, recurrent bacterial infections were detected, mainly at ear (> 10 episodes) and lung (4 pneumonias), leading to mild bronchiectasis (Fig. 2e). All infections were successfully treated with oral antibiotics, without hospitalization. IVIG replacement therapy (500 mg/kg q3w) was started in January 2016, which resulted in a decrease of the frequency of respiratory infections and in a marked improvement of patient’s health status.

### Hematological and Immunological Parameters

Laboratory monitoring revealed increased inflammatory markers (CRP, platelet count) and reduced hemoglobin in both patients (Supplementary Fig. S1). Immunological tests repeatedly revealed low-to-absent immunoglobulins and marked decrease of B cells in both patients, with T and NK cell counts repeatedly normal (Table 2). A comprehensive analysis of circulating B cells revealed their near complete absence in patient 1 and an overall decrease of all B cell subpopulations, a decreased response to polysaccharide vaccination, and the absence of autoantibodies in patient 2 (Table 2 and Supplementary Table S3). Bone marrow aspiration was once performed in patient 1, which revealed that B cell lineage represented 6.2% of total leukocytes and 28.4% of total lymphocytes, with the presence of all B cell stages. However, a reduction of the immature B cell stage compared with healthy controls was detected (Supplementary Fig. S2).

### Molecular Genetics

Genetic analyses identified rare candidate variants in both patients (Fig. 1b, c shows candidate filtering strategies; Supplementary Table S4 lists the gene variants detected in patient 1). Assuming a dominant inheritance pattern for the disease, patients only shared heterozygous variants at *PLCG2* gene (p.Leu845_Leu848del in Family 1 and p.Ala708Pro in Family 2) (Fig. 1d and Table 3). Additional investigations confirmed that they were novel, de novo, and germline *PLCG2* variants (Supplementary Table S5). According to the consensus joint recommendations of the American College of Medical Genetics and Genomics and the Association of Molecular Pathology [14], these two *PLCG2* variants were classified as pathogenic on the basis of different criteria including their de novo nature, their absence in healthy controls, their location in regulatory domains and in conserved residues of the protein (Fig. 1e–f), and the results of different bioinformatics and functional analyses (Table 3).

**Table 3:** Characteristics of *PLCG2* variants detected in enrolled patients

Patient	Chromosome position	Reference allele	Variant allele	Gene	Exon	cDNA alteration^1^	Predicted amino acid alteration	Population Genetics			Bioinformatics			
								1000 GP	ExAC	gnomAD	Polyphen-2 (Hum Div)	Mutation Taster	GERP Score	Evidence^2^
Pt 1	Chr 16: 81962181–81962192	TTAGGGTCTCTT	-	*PLCG2*	24	c.2533_2544del TTAGGGTCTCTT	p.(Leu845_Leu848del)	0	0	0	-	Pol	5.43	Pathogenic
Pt 2	Chr 16: 81953156	G	C	*PLCG2*	20	c.2122G>C	p.(Ala708Pro)	0	0	0	Prob Dam (0.997)	Dis Caus	4.99	Pathogenic

^1^RefSeq: NM_002661.3. ^2^On the basis of standards and guidelines proposed in the consensus recommendations of the American College of Medical Genetics and Genomics and the Association of Molecular Pathology [4].*Pt*, patient; *Chr*, chromosome; *1000 GP*, 1000 Genomes Project Phase 3; *ExAc*, Exome Aggregation Consortium; *gnomAD*, Genome Aggregation Database; *GERP*, Genomic Evolutionary Rate Profiling; *Prob Dam*, probably damaging; *Pol*, polymorphism; *Dis Caus*, disease causing

### Functional Characterization of PLCγ2 Variants

Analyses of Ca^2+^ flux in CD19^+^ cells after IgM crosslinking and ionomycin stimulation were performed in both patients using Fluo-3 flow cytometric assay. In patient 1, no firm conclusions were drawn from these analyses due to the nearly complete absence of circulating B cells (data not shown). By contrast, analyses performed in patient 2 revealed a significantly higher release of Ca^2+^ into the cytosol of B cells after IgM crosslinking stimulation than in cells from control subjects, whereas no significant differences were observed after ionomycin stimulation (Supplementary Fig. 3A-C).

**Fig. 3:**
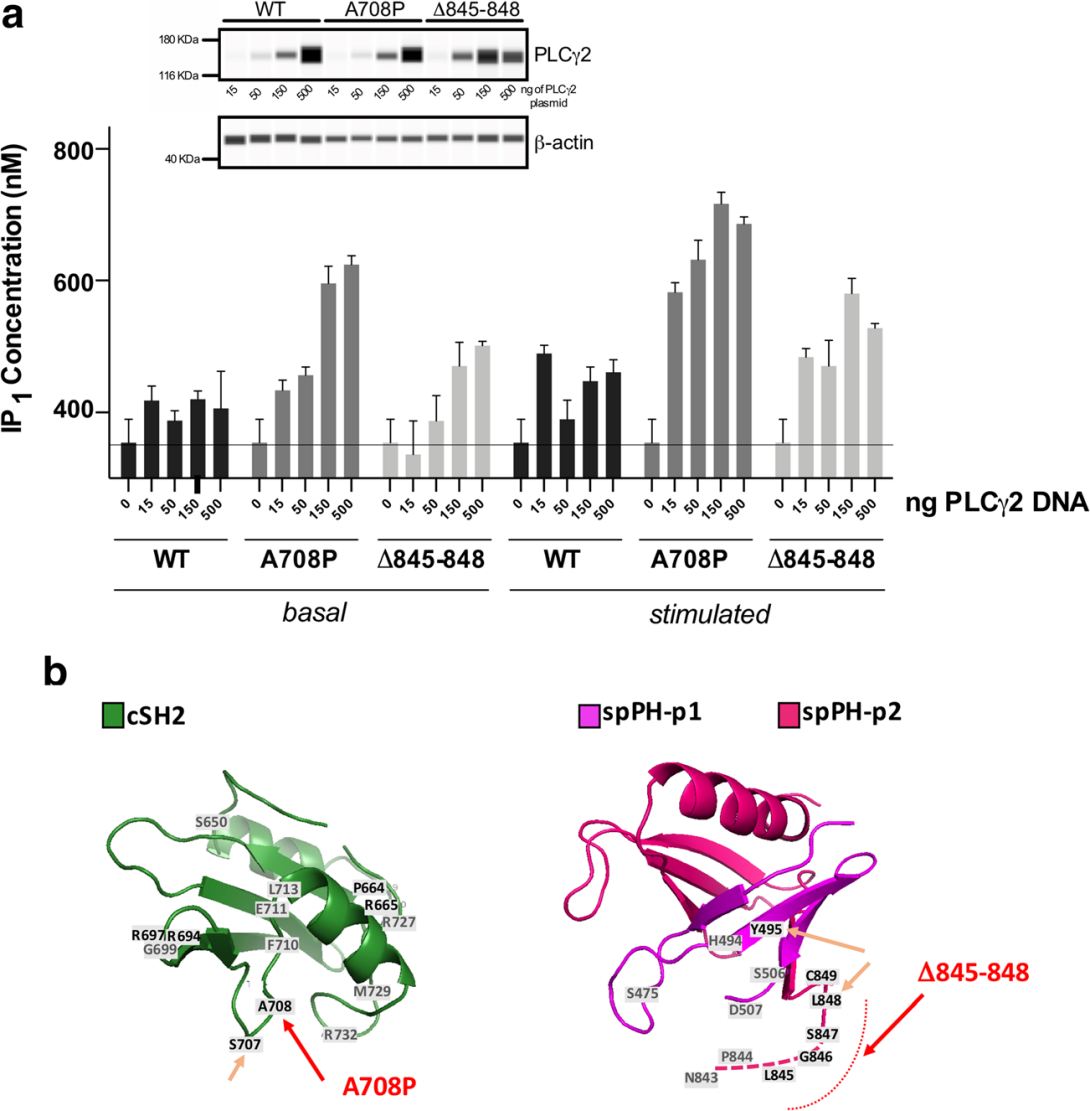
PLC activity analyses. **Panel a** The effect of p.Ala708Pro and p.Leu845_Leu848del variants on PLC activity was measured in transfected COS-7 cells under basal conditions (basal) or after stimulation by EGF (stimulated). Each data point represents the mean of triplicates and error bars indicate standard error of the mean. Expression levels of PLCγ2, corresponding to increasing concentrations of plasmids used for transfection, were measured using WES (top, inset). Further evaluation of the differences in PLC activities between the WT and variants was performed for the points with an equal protein expression. **Panel b** Position of p.Ala708 and p.Leu845-Leu848 segment (red arrows) is mapped on the structure of cSH2 and spPH domain, respectively. Positions of other mutations, reported for the main PLCγ1- and PLCγ2-linked pathologies that map to the same domains, are labeled using single letters and numbers corresponding to PLCγ2 sequence. Residues so far found to be mutated only in PLCγ1 are shown in gray. Other residues mutated in APLAID (p.Ser707 and p.Leu848) and Ali14 mice (p.Tyr495) are indicated by orange arrows

Further analysis of novel APLAID variants was performed in a standard PLC assay, similar to that previously used to measure the activity of PLAID and APLAID variants [6–8]. In this type of analysis, using model cell systems, the substrate is presented in native membranes and the production of inositol phosphates measured under basal or stimulated conditions. As shown in Fig. 3a, both variants have higher PLC activity compared with the wild type with the p.Ala708Pro substitution showing a greater increase in basal and stimulated activities. When analyzed in the context of CLL resistance to ibrutinib, p.Ala708Pro mutation had a pronounced effect [15], consistent with our observations.

### NLRP3-Inflammasome Activation and Cytokine Secretion

**Fig. 4:**
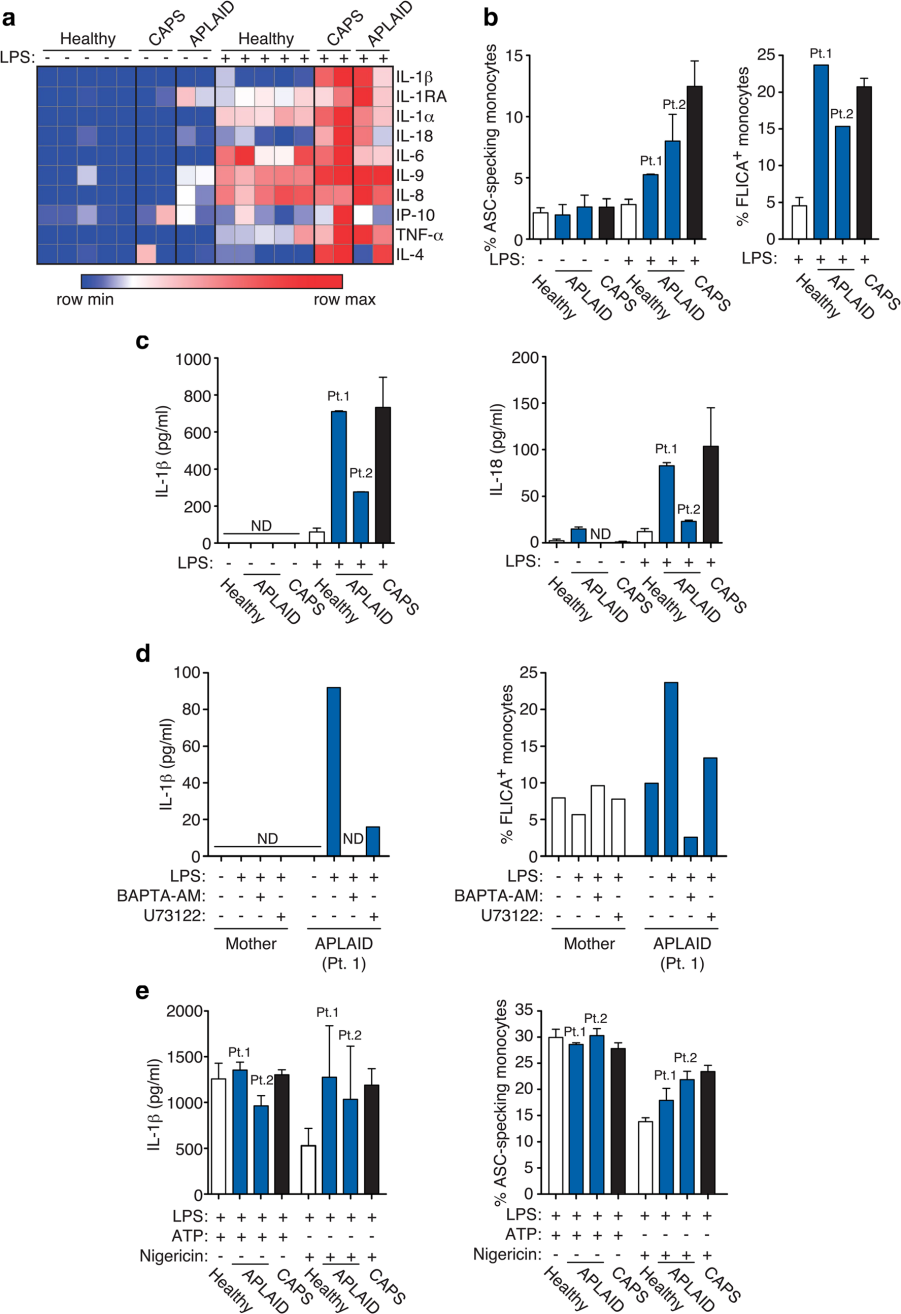
Involvement of NLRP3-inflammasome activation in sterile inflammation. **Panel a** Heat map of cytokine analysis from peripheral blood mononuclear cell (PBMCs) supernatants after LPS stimulation as indicated (1 μg/mL, 2 h) isolated from healthy controls (*n* = 5), patients with CAPS (heterozygous for p.Arg260Trp *NLRP3* mutation; *n* = 2) or APLAID carrying the heterozygous p.A708P (*n* = 1) or p.Leu845_Leu848del (*n* = 1) *PLCG2* variants. Representative of relative values of minimum and maximum concentrations measured per cytokine. **Panel b** Apoptosis-associated Speck-like protein containing a Caspase recruitment domain (ASC) speck forming monocytes by flow cytometry and active caspase-1 by YVAD-Fluorochrome Inhibitor of Caspases (FLICA) staining on monocytes after LPS stimulation as indicated (1 μg/mL, 2 h) isolated from healthy controls, patients with CAPS, or patients with APLAID. Showed results are representative of duplicate experiments. **Panel c** PBMCs IL-1β and IL-18 cytokine production at baseline and after LPS stimulation as indicated (1 μg/mL, 2 h) in healthy controls, patients with CAPS, and patients with APLAID. Showed results are representative of duplicate experiments. **Panel d** PBMCs IL-1β and percentage of monocytes stained for active caspase-1 by FLICA at baseline and after LPS stimulation (1 μg/mL, 2 h) in the presence or absence of BAPTA-AM (20 μM) or U73122 (2.5 μM) as indicated in APLAID patient 1 (p.Leu845_Leu848del *PLCG2* variant) and her healthy mother. Showed results are representative of experiments performed only once due to limited availability of samples. **Panel e** PBMCs IL-1β and ASC speck forming monocytes upon canonical NLRP3 activation by LPS priming (1 μg/mL, 2 h) followed by 30-min treatment with ATP (3 mM) or nigericin (10 μM) in healthy controls, patients with CAPS, and patients with APLAID. Showed results are representative of duplicate experiments. ND, not detected

PBMCs from APLAID patients showed an increased production of different cytokines after LPS treatment compared with healthy controls, which was similar to that detected in PBMCs from cryopyrin-associated periodic syndrome (CAPS) patients. The upregulated cytokines included proinflammatory cytokines such as TNF-α and different members of IL-1 family (IL-1α, IL-1β, IL-1Ra, IL-18) (Fig. 4a). The increased release of IL-1 cytokines occurred simultaneously to an increase of ASC speck formation and activation of caspase-1 on LPS-treated monocytes from APLAID and CAPS patients when compared with monocytes from healthy individuals (Fig. 4b). IL-1β and IL-18 release from APLAID patients’ PBMCs after LPS treatment was comparable to that of CAPS patients (Fig. 4c). The release of IL-1β and the activation of caspase-1 induced by LPS in APLAID PBMCs were reduced when intracellular calcium was chelated with BAPTA-AM or when the widely used PLC inhibitor U73122 was used (Fig. 4d). Canonical activation of the NLRP3-inflammasome by adding ATP or nigericin after LPS priming resulted in an equal formation of intracellular ASC specks in monocytes and similar release of IL-1β in samples from healthy donors, APLAID patients, and CAPS patients (Fig. 4e). Collectively, these results suggest an over-activation of the alternative NLRP3 inflammasome pathway in monocytes from APLAID patients, which could be enhanced by the elevated levels of intracellular calcium associated with the increased activity of mutated PLCγ2.

## Discussion

The patients here described were initially suspected of suffering from a rare disease combining humoral immunodeficiency and inflammatory manifestations. Previous genetic studies did not identify defects in different genes causing monogenic antibody deficiencies or AIDs [1, 16]. Due to strong similarities, we hypothesized that the patients’ disease might be a consequence of defects in the same gene. Our further genetic studies revealed that each patient carried a novel, de novo heterozygous *PLCG2* pathogenic variant. Furthermore, our extensive characterization of clinical manifestations, properties of immune cells, and changes in function of the encoded enzyme, PLCγ2, support a definitive diagnosis of PLCγ2-associated antibody deficiency and immune dysregulation syndrome, designated as APLAID.

Previously, only 4 patients from three families have been diagnosed with APLAID [7–9] and have been found to share some of the manifestations with *Ali5* and *Ali14* mouse strains that carry *gain-of-function PLCG2* mutations [17, 18]. Another two families carrying the same *PLCG2* variant in the C2 domain of the protein have been recently described during manuscript reviewing [19]. The two unrelated patients described in our study enable us to make more extensive comparisons and highlight the key changes that characterize the human syndrome and parallels with the mouse models. These include severe inflammatory lesions at skin, ocular, and joints mediated by non-lymphoid hematopoietic cells and variable degree of immunodeficiency. With regard to the immune defects, all known APLAID patients exhibited variable degrees of B cell lymphopenia and antibody deficiency, with no apparent impairment of T and NK functions (Table 1). Patient 1 carrying the p.Leu845_Leu848 *PLCG2* deletion showed the complete absence of B cells and circulating immunoglobulins, an immune phenotype compatible with a rare form of non-X-linked agammaglobulinemia [16] and quite similar to the immune phenotype of the APLAID patients carrying the missense p.Leu848Pro *PLCG2* variant [8, 9]. The humoral immunodeficiency of this patient (patient 1) was clinically characterized by severe and recurrent bacterial infections, structural lung lesions, recurrent episodes of acute hemoptysis, and the requirement of surgical lung lobe resection. To our knowledge, this patient represents the severest APLAID patient described to date and clearly expands the diversity of the immune deficiency of this disease toward phenotypes compatible with dominantly inherited agammaglobulinemia. Since allogeneic hematopoietic stem cell transplantation has been proposed as a curative option in severe PID [20, 21], a major unsolved question is whether this particular patient would have benefited from this therapeutic approach at a younger age, even with the causative genetic defect not elucidated. With regard to patient 2 who carried the missense p.Ala708Pro *PLCG2* variant, his immunological phenotype (low IgM and B cell count and decreased circulating class-switched memory B cells) could be classified as mild-to-moderate and displayed marked similarities with the phenotype of the first described APLAID family carrying the missense p.Ser707Tyr *PLCG2* variant [7]. The B cell disturbances observed in patients with APLAID are variable ranging from patients displaying low circulating B cells to patients with absent B cells. *PLCG2* was proposed as a good candidate gene to explain some B cell deficiencies in humans because the mouse model of PLCγ2 deficiency showed a decrease of mature B cell and a block in the pro-B cell differentiation [22]. However, the human deficiency of PLCγ2 has not been yet described. By contrast, monoallelic *PLCG2* mutations are associated with variable humoral immune deficiency and immune dysregulation in PLAID and APLAID syndromes [6–9]. The few available data about the defects of the B cell lineage in APLAID patients do not permit to drawn firm conclusions about the precise molecular mechanisms underlying the humoral deficiency in these patients. Considering the relevant role of PLCγ2 in multiple signal transduction pathways in B cells and the *gain-of-function* nature of *PLCG2* variants detected in APLAID syndrome, a mechanism that may explain the immune phenotype observed and that should be further investigated is the deletion and/or functional anergy of specific B cell progenitors due to enhanced cellular signaling.

We were particularly interested in sterile inflammatory lesions and their molecular basis. From a clinical perspective, the cutaneous manifestations were the most prominent in both of our patients (patient 1 and 2) since the onset of their diseases as have been also described for the other four published APLAID patients [7–9]. These manifestations were initially vesicular and blistering lesions that subsequently evolved to the destruction of elastin fibers leading to large areas of *cutis laxa* in both patients, a phenomenon also described in the APLAID patient with the p.Leu848Pro *PLCG2* variant [8]. Later in life both patients also developed severe eye inflammation, mainly affecting conjunctiva and episclera. The beneficial effect of topic anakinra in the ocular lesions in PLAID [23] suggests the potential combination of topical ocular treatments to the systemic therapies that both patients are already receiving.

We have further investigated the molecular mechanisms underlying sterile inflammation in APLAID. We found that the novel PLCγ2 variants here identified were able to induce activation of the NLRP3 inflammasome, with ASC aggregation, caspase-1 activation, and IL-1β and IL-18 release in monocytes. Previous evidence suggested a potential connection between hypermorphic *PLCG2* mutations and NLRP3-inflammasome activation throughout the increase of intracellular calcium [7, 16, 24, 25]. Consequently, it is possible that in the patients described here, the sterile inflammation manifestations could be also linked to NLRP3-inflammasome. Our results showed that canonical activation pathway of NLRP3-inflammasome in patient’s monocytes did not result in an increase of IL-1β release or ASC-specking as observed for healthy monocytes. Therefore, the inflammasome pathway that is potentiated in APLAID might be related to the alternative NLRP3 activation pathway, similar to that described in human monocytes upon LPS activation [26], rather than the canonical pathway that requires ATP or nigericin as second stimuli after LPS priming [27].

Finally, our identification of two new pathogenic variants in *PLCG2* linked to APLAID (p.Ala708Pro and p.Leu845_Leu848) expands the range of genetic changes that cause this syndrome. Consistent with previous studies [7–9], we show that the new *PLCG2* variants have higher PLC activity compared with the wild type. Interestingly, p.Ala708Pro *PLCG2* variant has been also discovered in ibrutinib-resistant chronic lymphocytic leukemia (CLL) in the form of somatic variant [28–30]. Two other somatic *PLCG2* variants detected in resistant CLL, p.Ser707Tyr and p.Asp993Gly, are also shared with immune disorders in APLAID [7] and *Ali5* mice [17], respectively. More broadly, the new variants map to distinct regions in the cSH2 domain, and spPH domain and its vicinity, the regions that appear to harbor mutations across different PLCγ-linked pathologies with higher frequencies (Fig. 3b). Therefore, the molecular mechanisms that lead to an increase in PLC activity in diverse pathologies could be similar. Considerable supporting evidence is consistent with a model where PLCγ2 is kept in an inactive state by extensive intramolecular interactions between the regulatory domains (including cSH2 and spPH domains) and the PLC-core domains [11, 31]. Release of this autoinhibition by physiological stimulation (via phosphorylation) or by mutations represents an important step leading to an increase in PLC activity. Therefore, our data showing that the novel variants are hypermorphic and their position in the protein highlight particular structural features within the regulatory array that are the key regions involved in autoinhibition.

In conclusion, from the perspective of clinical practice, the detection of a novel, de novo hypermorphic *PLCG2* pathogenic variant in each of the two patients displaying early-onset severe skin and eye inflammation and humoral immunodeficiency supports their definitive diagnosis of the extremely rare APLAID syndrome. Moreover, the patients’ immunological features expand the APLAID diversity toward most severe phenotypes than previously described including complete B cell depletion and agammaglobulinemia inherited as a dominant trait, which should be considered when evaluating patients with severe deficiencies of antibody production.

## Electronic supplementary material

Supplementary Data include 3 supplementary figures and 6 supplementary tables and can be found with this article online.

ESM 1 (DOC 279 kb)

ESM 2 (PNG 873 kb)

High Resolution Image (TIFF 99.5 kb)
