# Supplementary material for: Severe Autoinflammatory Manifestations and Antibody Deficiency Due to Novel Hypermorphic PLCG2 Mutations
Source: J Clin Immunol. 2020 Jul 15;40(7):987–1000. doi: 10.1007/s10875-020-00794-7 (PMC7505877; doi:10.1007/s10875-020-00794-7)
Supplement: Supplementary file 1 — (DOC 279 kb) [file 10875_2020_794_MOESM1_ESM.doc]

**Supplementary Data**

**Severe Autoinflammatory Manifestations and Antibody Deficiency Due to Novel Hypermorphic *PLCG2* Mutations**

Andrea Martín-Nalda MD^1,2,*^, Claudia Fortuny MD PhD^3,4,*^, Lourdes Rey MD^5^, Tom D. Bunney PhD^6^, Laia Alsina MD PhD^4,7,8^, Ana Esteve-Solé PhD^4,7,8^, Daniel Bull PhD^9^, Maria Carmen Anton AS^10^, María Basagaña MD PhD^11^, Ferran Casals PhD^12^, Angela Deyá MD PhD^4,7,8^, Marina García-Prat^1,2^, Ramon Gimeno MD PhD^13^, Manel Juan MD PhD^10,14,15^, Helios Martinez-Banaclocha PhD^16^, Juan J Martinez-Garcia PhD^16^, Anna Mensa-Vilaró PhD^10^, Raquel Rabionet PhD^4,17^, Nieves Martin-Begue MD^18^, Francesc Rudilla PhD^19,20^, Jordi Yagüe MD PhD^10,14,15^, Xavier Estivill MD PhD^21^, Vicente García-Patos MD PhD^22^, Ramon M. Pujol MD PhD^23^, Pere Soler-Palacín MD PhD^1,2,24^, Matilda Katan PhD^6^, Pablo Pelegrín PhD^16,**^, Roger Colobran PhD^2,25,26,**^, Asun Vicente MD^27,**^, Juan I. Arostegui MD PhD^10,14,15,**^

**CONTENTS**

1. **Supplementary Tables**
2. **Legends of Supplementary Figures**

**SUPPLEMENTARY TABLES**

**Supplementary Table S1.** **List of employed antibodies**.

| **Antibody** | **Clone** | **Fluorochromes** | **Company** | **Catalogue Number** |
| --- | --- | --- | --- | --- |
| anti-human IgG | H2 | Phycoerythrin (PE) | Beckman Coulter | Cat#733173 |
| anti-human IgA | Polyclonal | Alexa Fluor®647 | Jackson ImmunoResearch Labs | Cat#109-605-011 |
| anti-human IgM | UHB | Fluorescein isothiocyanate (FITC) | Beckman Coulter | Cat#733152 |
| anti-human IgM | G20-127 | PerCP-Cy™5.5 | BD Biosciences | Cat#561285 |
| anti-human IgM | G20-127 | Brilliant™ Violet 421 | BD Horizon | Cat#562618 |
| anti-human IgD | IADB6 | Fluorescein isothiocyanate (FITC) | Beckman Coulter | Cat#735993 |
| anti-human IgD | IADB6 | Phycoerythrin (PE) | Beckman Coulter | Cat#736000 |
| anti-human TCRαβ | WT31 | Fluorescein isothiocyanate (FITC) | BD Biosciences | Cat#340883 |
| anti-human TCRγδ | 11F2 | Phycoerythrin (PE) | BD Biosciences | Cat#340887 |
| anti-human CD3 | UCHT1 | Allophycocyanin (APC)-Alexa Fluor®750 | BD Biosciences | Cat#557943 |
| anti-human CD3 | SK7 | Brilliant™ Violet 421 | BD Horizon | Cat#563798 |
| anti-human CD3 | SK7 | Allophycocyanin (APC)-Cy™7 | BD Biosciences | Cat#341110 |
| anti-human CD3 | SK7 | Allophycocyanin (APC)-H7 | BD Biosciences | Cat#641415 |
| anti-human CD4 | 13B8.2 | Fluorescein isothiocyanate (FITC) | Beckman Coulter | Cat#A07750 |
| anti-human CD4 | RPA-T4 | Brilliant™ Violet 421 | BD Biosciences | Cat#562425 |
| anti-human CD5 | OX19 | Fluorescein isothiocyanate (FITC) | Beckman Coulter | Cat#IM3060 |
| anti-human CD8 | RPA-T8 | PerCP-Cy™5.5 | BD Pharmingen | Cat#560662 |
| anti-human CD8 | SFCI21Thy2D3-(T8) | Phycoerythrin-Cyanine5.5 | Beckman Coulter | Cat#A99019 |
| anti-human CD14 | 61D3 | Allophycocyanin (APC) | TONBO Biosciences | Cat#20-0149 |
| anti-human CD16 | 3G8 | Phycoerythrin (PE) | Beckman Coulter | Cat#A07766 |
| anti-human CD19 | SJ25C1 | Phycoerythrin (PE)-Cy™7 | BD Biosciences | Cat#341113 |
| anti-human CD19 | SJ25C1 | Brilliant™ Violet 510 | BD Horizon | Cat#562947 |
| anti-human CD19 | SJ25C1 | Allophycocyanin (APC)-Cy™7 | BD Biosciences | Cat#348794 |
| anti-human CD19 | SJ25C1 | Allophycocyanin (APC)-H7 | BD Biosciences | Cat#641395 |
| anti-human CD20 | B9E9(HRC20) | Allophycocyanin (APC) | Beckman Coulter | Cat#A21693 |
| anti-human CD20 | BL13 | Phycoerythrin (PE) | Beckman Coulter | Cat#A32536 |
| anti-human CD21 | B-ly4 | Fluorescein isothiocyanate (FITC) | BD Biosciences | Cat#561372 |
| anti-human CD21 | BL13 | Phycoerythrin (PE) | Beckman Coulter | Cat#A32536 |
| anti-human CD24 | ML5 | PerCP-Cy™5.5 | BD Pharmingen | Cat#561647 |
| anti-human CD27 | M-T271 | Allophycocyanin (APC) | BD Pharmingen | Cat#558664 |
| Anti.human CD33 | AC104.3E3 | Allophycocyanin (APC)-vio 770 | Miltenyi Biotech | Cat#130-101-149 |
| anti-human CD38 | HB-7 | Phycoerythrin (PE)-Cy™7 | BD Biosciences | Cat#335825 |
| anti-human CD38 | LS198-4-3 | Phycoerythrin-Cyanine 7 | Beckman Coulter | Cat#A54189 |
| anti-human CD45 | J33 | Allophycocyanin (APC) | Beckman Coulter | Cat#IM2473 |
| anti-human CD45 | 2D1 | Allophycocyanin (APC)-H7 | BD Biosciences | Cat#641417 |
| anti-human CD45 | HI30 | Phycoerythrin (PE)-Cy™7 | BD Pharmingen | Cat#557748 |
| anti-human CD45 | HI30 | Brilliant™ Violet 510 | BD Horizon | Cat#563204 |
| anti-human CD45RA | L48 | Phycoerythrin (PE)-Cy™7 | BD Biosciences | Cat#337186 |
| anti-human CD45RO | UCHL-1 | Allophycocyanin (APC) | BD Biosciences | Cat#340438 |
| anti-human CD56 | N901 (NKH-1) | Phycoerythrin-Cyanine 7 | Beckman Coulter | Cat#A21692 |
| anti-human CD57 | NC1 | Fluorescein isothiocyanate (FITC) | Beckman Coulter | Cat#IM0466U |
| anti-human CD62L | DREG56 | Fluorescein isothiocyanate (FITC) | Beckman Coulter | Cat#IM1231U |

**Supplementary Table S2. List of Primers used in the Sanger Method of DNA Sequencing of the *PLCG2* Gene**. Capital fonts indicate nucleotides of exonic regions and lower-case fonts indicate nucleotides of intronic regions.

| **Exon** | **Sense primer** | **Anti-sense primer** |
| --- | --- | --- |
| 20 | 5’-ggttgtatctaatcagtagggtttg | 5’-TAGCGCAGTCTCATCTTTCG |
| 24 | 5’- ctgctaaacggtgtgctttg | 5’-agccacctccctgtgtagg |

**Supplementary Table S3.** **B Cell Subpopulations Analyses in Patient 2**. ^1^Figures in brackets indicate the normal 5^th^ and 95^th^ percentiles of each subpopulation adjusted per age (data obtained from Duchamp M, et al. Immun Inflamm Dis 2014; 2: 131-140). ^2^Figures in brackets indicate the normal 25^th^ and 75^th^ percentiles of each subpopulation adjusted per age (data obtained from Morbach H, et al. Clin Exp Immunol 2010; 162: 271-279). Abbreviations: m, months; y, years.

| **B Cell Subpopulations** | **Cell Surface Markers** | **5y 6m** (Total B cells 31/μL) | |
| --- | --- | --- | --- |
|  |  | **Percentage** | **Total (cels/μL)** |
| Naive B cells^1^ | CD19^+^ CD27^-^ IgD^+^ IgM^+^ | 45.8 (59.7-88.4) | 14.2 (203-648) |
| Non-switched Memory B cells / Marginal zone B cells^1^ | CD19^+^ CD27^+^ IgD^+^ | 5.0 (3.1-18.0) | 1.6 (7-91) |
| Switched Memory B cells^1^ | CD19^+^ CD27^+^ IgD^-^ | 10.3 (2.9-17.4) | 3.2 (11-103) |
| Plasmablasts^2^ | CD19^+^ CD38^hi^ IgM^-^ | 10.6 (0.7-3.5) | 3.3 (2-12) |
| Innate-like B cells^2^ | CD19^+^ CD38^low^ CD21^low^ | 12.5 (0.9-3.5) | 3.9 (3-4) |

**Supplementary Table S4. Rare Gene Variants (Allele Frequency <0.001) Detected in Patient 1**. Abbreviations: Chr, chromosome; cDNA, complementary DNA; 1000 GP, 1000 Genomes Project Phase 3; ExAc, Exome Aggregation Consortium; gnomAD, Genome Aggregation Database; SIFT, Sorting Intolerant from Tolerant.

| Chr | Coordinate | Gene | RefSeq | cDNA alteration | Predicted amino acid alteration | dbSNP  ID | ExAC |  | Bioinformatics | |
| --- | --- | --- | --- | --- | --- | --- | --- | --- | --- | --- |
|  |  |  |  |  |  |  |  |  | SIFT  (Score) | Polyphen  (Score) |
| 1 | 16354354 | CLCNKA | NM_004070.3 | c.820G>A | p.Asp274Asn | rs368824021 | 0.000133 |  | Deleterious  (0) | Probably damaging  (0.949) |
| 1 | 27023450 | ARID1A | NM_006015.4 | c.557_559delGCG | p.Gly187del | - | 0 |  | n.a. | n.a. |
| 1 | 161594399 | FCGR3B | NM_001244753.1 | c.716C>T | p.Ser239Phe | - | 0 |  | Tolerated  (1) | Benign  (0.001) |
| 1 | 171177988 | FMO2 | NM_001460.2 | c.1312G>A | p.Glu438Lys | rs145876121 | 0.0006756 |  | Deleterious  (0.01) | Probably damaging  (0.967) |
| 1 | 197102534 | ASPM | NM_018136.4 | c.2365A>G | p.Ile789Val | - | 0.00004988 |  | Tolerated  (0.25) | Possibly damaging  (0.845) |
| 1 | 205898454 | SLC26A9 | NM_134325.2 | c.748C>T | p.His250Tyr | rs143411715 | 0.0004453 |  | Deleterious  (0.02) | Benign  (0.348) |
| 1 | 235972391 | LYST | NM_000081.2 | c.1727G>A | p.Gly576Asp | - | 0 |  | Deleterious  (0.04) | Probably damaging  (0.999) |
| 1 | 237527679 | RYR2 | NM_001035.2 | c.309+7C>T | - | - | 0.0007640 |  | n.a. | n.a. |
| 2 | 20201776 | MATN3 | NM_002381.4 | c.982T>C | p.Ser328Pro | - | 0 |  | Deleterious  (0.02) | Probably damaging  (0.924) |
| 2 | 71742765 | DYSF | NM_001130987.1 | c.772G>A | p.Val258Met | rs150345121 | 0.0001156 |  | Deleterious  (0) | probably_damaging  (0.981) |
| 2 | 73613031 | ALMS1 | NM_015120.4 | c.36_41delGGAGGAinsGGAGGAGGA | p.Glu11dup | - | 0 |  | n.a. | n.a. |
| 2 | 169791823 | ABCB11 | NM_003742.2 | c.2927A>G | p.Gln976Arg | rs199940188 | 0.0005051 |  | Tolerated  (0.58) | Benign  (0.003) |
| 2 | 170063446 | LRP2 | NM_004525.2 | c.6784C>T | p.Arg2262Cys | - | 0.00003295 |  | Deleterious  (0.04) | Benign  (0.269) |
| 2 | 216285504 | FN1 | NM_212482.1 | c.1567A>C | p.Ile523Leu | rs200567544 | 0.0005357 |  | Tolerated  (0.76) | Possibly damaging  (0.71) |
| 2 | 227919393 | COL4A4 | NM_000092.4 | c.2777A>G | p.Glu926Gly | - | 0 |  | Tolerated  (0.36) | Benign  (0.067) |
| 2 | 228176570 | COL4A3 | NM_000091.4 | c.4997T>A | p.Met1666Lys | - | 0 |  | Deleterious  (0) | Probably damaging  (0.996) |
| 3 | 53783321 | CACNA1D | NM_000720.2 | c.3401A>G | p.Asn1134Ser | - | 0 |  | Tolerated  (0.1) | Benign  (0.014) |
| 3 | 58080583 | FLNB | NM_001164317.1 | c.808A>G | p.Met270Val | rs145036794 | 0.0005601 |  | Tolerated  (0.53) | Benign  (0.009) |
| 3 | 130150452 | COL6A5 | NM_153264.5 | c.5392G>A | p.Gly1798Arg | rs569432794 | 0.0001009 |  | Deleterious  (0) | Probably damaging  (1) |
| 3 | 140281707 | CLSTN2 | NM_022131.2 | c.2267A>G | p.Asn756Ser | rs766788653 | 0.00008246 |  | Tolerated  (0.49) | Benign  (0.205) |
| 3 | 164783041 | SI | NM_001041.3 | c.807+8A>G | - | - | 0.00001684 |  | n.a. | n.a. |
| 4 | 96025724 | BMPR1B | NM_001256793.1 | c.233+6T>G | - | - | 0 |  | n.a. | n.a. |
| 5 | 5200279 | ADAMTS16 | NM_139056.2 | c.1348T>G | p.Cys450Gly | - | 0 |  | Deleterious  (0) | Probably damaging  (1) |
| 5 | 6623405 | NSUN2 | NM_017755.5 | c.466-8delT | - | - | 0 |  | n.a. | n.a. |
| 6 | 16327903 | ATXN1 | NM_001128164.1 | c.638_639insTCA | p.Gln213insHis | - | 0 |  | n.a. | n.a. |
| 6 | 16327915 | ATXN1 | NM_001128164.1 | c.624_626dupGCA | p.Gln208dup | - | 0 |  | n.a. | n.a. |
| 6 | 36270090 | PNPLA1 | NM_001145717.1 | c.1228T>G | p.Ser410Ala | - | 0.000008261 |  | Deleterious  (0.03) | Benign  (0.093) |
| 6 | 56506814 | DST | NM_015548.4 | c.347G>A | p.Arg116His | - | 0.00002487 |  | Deleterious  (0.01) | Possibly damaging  (0.548) |
| 6 | 76572397 | MYO6 | NM_004999.3 | c.1631C>T | p.Thr544Ile | rs371944427 | 0.00007419 |  | Deleterious  (0) | Probably damaging  (0.993) |
| 6 | 101315799 | ASCC3 | NM_006828.2 | c.75A>C | p.Glu25Asp | - | 0 |  | Tolerated  (0.38) | Benign  (0.001) |
| 6 | 135520156 | MYB | NM_001130173.1 | c.1677C>A | p.Asp559Glu | rs143010463 | 0.0002407 |  | Tolerated  (1) | Benign  (0.043) |
| 7 | 286468 | FAM20C | NM_020223.3 | c.951_952insGACAGGTGAGCCCTTCCTTCCTCCCTCCATCCGC | p.Ile320Ter | - | 0 |  | n.a. | n.a. |
| 7 | 77789464 | MAGI2 | NM_012301.3 | c.2723C>G | p.Pro908Arg | rs150080418 | 0.0002976 |  | Tolerated  (0.33) | Benign  (0.008) |
| 7 | 99030926 | ATP5J2-PTCD1 | NM_001198879.1 | c.716A>G | p.Lys239Arg | rs111989725 | 0.0002636 |  | Deleterious  (0.01) | Probably damaging  (0.983) |
| 7 | 103124188 | RELN | NM_005045.3 | c.10093G>A | p.Val3365Ile | rs115035120 | 0.0009225 |  | Tolerated  (0.51) | Probably damaging  (0.973) |
| 7 | 122635669 | TAS2R16 | NM_016945.2 | c.20C>T | p.Thr7Ile | - | 0.00001809 |  | Deleterious  (0.01) | Possibly damaging  (0.695) |
| 7 | 151664354 | GALNTL5 | NM_145292.3 | c.23G>T | p.Gly8Val | - | 0.00002476 |  | Tolerated  (0.19) | Benign  (0.003) |
| 8 | 28574669 | EXTL3 | NM_001440.2 | c.1093G>C | p.Glu365Gln | - | 0 |  | Tolerated  (0.5) | Possibly damaging  (0.675) |
| 8 | 100115349 | VPS13B | NM_017890.4 | c.580+1G>A | - | - | 0 |  | n.a. | n.a. |
| 9 | 32986031 | APTX | NM_001195248.1 | c.526-25_526-4delGTTTTTTTTTTTGTTTTTTTTTinsT | - | - | 0 |  | n.a. | n.a. |
| 9 | 101599420 | GALNT12 | NM_024642.4 | c.1202G>A | p.Arg401His | - | 0 |  | Tolerated  (0.43) | Benign  (0.001) |
| 9 | 101602410 | GALNT12 | NM_024642.4 | c.1339G>A | p.Gly447Arg | rs376441206 | 0.00009884 |  | Deleterious  (0) | Probably damaging  (1) |
| 9 | 135946915 | CEL | NM_001807.3 | c.2035G>T | p.Ala679Ser | - | 0 |  | Tolerated  (1) | Benign  (0) |
| 9 | 135946921 | CEL | NM_001807.3 | c.2041C>G | p.Pro681Ala | rs490954 | 0.0003658 |  | Tolerated  (1) | Benign  (0) |
| 9 | 136570084 | SARDH | NM_007101.3 | c.1540C>T | p.Arg514Ter | rs140559739 | 0.0002555 |  | n.a. | n.a. |
| 10 | 55780122 | PCDH15 | NM_001142763.1 | c.2596G>A | p.Val866Met | rs142512524 | 0.0005767 |  | Tolerated  (0.09) | Probably damaging  (0.998) |
| 10 | 116919795 | ATRNL1 | NM_207303.2 | c.830-6T>A | - | rs368819999 | 0.0007799 |  | n.a. | n.a. |
| 11 | 6411935 | SMPD1 | NM_000543.4 | c.108_113delGCTGGC | p.Ala48_Leu49del | rs3838786 | 0.00002701 |  | n.a. | n.a. |
| 11 | 36596718 | RAG1 | NM_000448.2 | c.1864G>A | p.Ala622Thr | rs148380512 | 0.0001401 |  | Deleterious  (0) | Probably damaging  (0.956) |
| 11 | 60886423 | CD5 | NM_014207.3 | c.437C>T | p.Pro146Leu | rs139537878 | 0.0004496 |  | Tolerated  (0.27) | Benign  (0.167) |
| 12 | 346407 | SLC6A13 | NM_016615.4 | c.613C>T | p.Arg205Cys | rs199825876 | 0.0003555 |  | Deleterious  (0) | Probably damaging  (0.984) |
| 12 | 12871129 | CDKN1B | NM_004064.3 | c.356T>C | p.Ile119Thr | rs142833529 | 0.0004723 |  | Tolerated  (0.29) | Benign  (0.011) |
| 12 | 53038906 | KRT2 | NM_000423.2 | c.1817C>T | p.Ser606Phe | rs746933813 | 0.0000412 |  | Deleterious  (0) | Possibly damaging  (0.879) |
| 12 | 56115129 | RDH5 | NM_002905.3 | c.161G>A | p.Arg54Gln | rs200548164 | 0.00006719 |  | Tolerated  (0.17) | Benign  (0.138) |
| 12 | 57498959 | STAT6 | NM_003153.4 | c.976G>C | p.Val326Leu | - | 0 |  | Tolerated  (1) | Benign  (0.001) |
| 12 | 57604255 | LRP1 | NM_002332.2 | c.12746C>T | p.Ser4249Phe | - | - |  | Tolerated  (0.16) | Probably damaging  (0.991) |
| 12 | 133446352 | CHFR | NM_001161344.1 | c.472G>A | p.Asp158Asn | - | 0.00004705 |  | Tolerated  (0.09) | Benign  (0.002) |
| 13 | 23912948 | SACS | NM_014363.4 | c.5065_5066delAG | p.Ser1689CysfsTer12 | - | 0 |  | n.a. | n.a. |
| 13 | 32835887 | FRY | NM_023037.2 | c.7551G>T | p.Glu2517Asp | rs144509680 | 0.00007457 |  | Tolerated  (0.33) | Benign  (0.006) |
| 13 | 103718396 | SLC10A2 | NM_000452.2 | c.162_203dupAATCAAGAAATTTCTAGGGCACATAAAGCGGCCGTGGGGCAT | p.Ile68_Cys69insIleLysLysPheLeuGlyHisIleLysArgProTrpGlyIle | - | 0 |  | n.a. | n.a. |
| 14 | 23858271 | MYH6 | NM_002471.3 | c.3979-8delC | - | - | 0 |  | n.a. | n.a. |
| 14 | 64685985 | SYNE2 | NM_182914.2 | c.19717A>G | p.Lys6573Glu | - | 0.000008258 |  | Deleterious  (0.01) | Possibly damaging  (0.846) |
| 14 | 74968282 | LTBP2 | NM_000428.2 | c.5182G>A | p.Gly1728Ser | - | 0.000008368 |  | Tolerated  (0.82) | Benign  (0.014) |
| 14 | 75022240 | LTBP2 | NM_000428.2 | c.987G>T | p.Gln329His | rs201070032 | 0.0000831 |  | Tolerated  (0.33) | Possibly damaging  (0.539) |
| 14 | 105175957 | INF2 | NM_022489.3 | c.2053A>G | p.Ile685Val | rs199526439 | 0.0001686 |  | Tolerated  (0.21) | Benign  (0.034) |
| 15 | 41803368 | LTK | NM_002344.5 | c.988_990delGGC | p.Gly330del | - | 0 |  | n.a. | n.a. |
| 15 | 78882257 | CHRNA5 | NM_000745.3 | c.524C>T | p.Thr175Met | - | 0.00001648 |  | Deleterious  (0.02) | Probably damaging  (0.999) |
| 15 | 90320120 | MESP2 | NM_001039958.1 | c.533_558delAGGGGCAGGGGCAAGGGCAGGGGCAGinsAGGGGCAGGGGCAA | p.Gly183_Gln186del | - | 0 |  | n.a. | n.a. |
| 15 | 91025498 | IQGAP1 | NM_003870.3 | c.3540G>T | p.Glu1180Asp | - | 0.00001648 |  | Tolerated  (0.07) | Benign  (0.014) |
| 15 | 100252709 | MEF2A | NM_005587.2 | c.1234_1239delCAGCAG | p.Gln412_Gln413del | - | 0 |  | n.a. | n.a. |
| 16 | 1279041 | TPSB2 | NM_024164.5 | c.523T>C | p.Ser175Pro | - | 0 |  | Deleterious  (0) | Possibly damaging  (0.555) |
| 16 | 1279945 | TPSB2 | NM_024164.5 | c.8A>G | p.Asn3Ser | - | 0 |  | Tolerated  (0.54) | Benign  (0) |
| 16 | 70551524 | COG4 | NM_015386.2 | c.369+5T>C | - | - | 0 |  | n.a. | n.a. |
| 16 | 81962180 | PLCG2 | NM_002661.3 | c.2533_2544delTTAGGGTCTCTT | p.Leu845_Leu848delinsdel |  | 0 |  | n.a. | n.a. |
| 17 | 34171710 | TAF15 | NM_139215.2 | c.1408_1431delGGCTATGGAGGAGACCGAGGAGGT | p.Gly470_Gly477delinsdel | - | 0 |  | n.a. | n.a. |
| 17 | 38975103 | KRT10 | NM_000421.3 | c.1654_1683dupAGCTCCGGCGGCGGATACGGCGGCGGCAGC | p.Ser552_Ser561dup | - | 0 |  | n.a. | n.a. |
| 19 | 4157162 | CREB3L3 | NM_032607.1 | c.327_328insA | p.Pro110ThrfsTer36 | - | 0 |  | n.a. | n.a. |
| 19 | 41351305 | CYP2A6 | NM_000762.5 | c.1055T>C | p.Met352Thr | rs143841823 | 0.0004124 |  | Tolerated  (1) | Benign  (0.012) |
| 19 | 49657710 | HRC | NM_002152.2 | c.782_784delATG | p.Asp261del | rs66501117 | 0.0002104 |  | n.a. | n.a. |
| 19 | 55253521 | KIR2DL3 | NM_015868.2 | c.166C>G | p.Gln56Glu | rs35719984 | 0.00003781 |  | Tolerated  (0.76) | Benign  (0) |
| 19 | 55253552 | KIR2DL3 | NM_015868.2 | c.197T>A | p.Phe66Tyr | - | 0 |  | Tolerated  (0.93) | Benign  (0.005) |
| 19 | 55286769 | KIR2DL1 | NM_014218.2 | c.523C>A | p.Pro175Thr | rs74456429 | 0.00011 |  | Tolerated  (0.18) | Benign  (0.001) |
| 19 | 55286796 | KIR2DL1 | NM_014218.2 | c.550G>A | p.Asp184Asn | rs147072532 | 0.00009025 |  | Tolerated  (0.37) | Benign  (0.012) |
| 19 | 55286854 | KIR2DL1 | NM_014218.2 | c.608A>G | p.His203Arg | - | 0 |  | Tolerated  (1) | Benign  (0) |
| 19 | 55295215 | KIR2DL1 | NM_014218.2 | c.997A>G | p.Thr333Ala | rs2756923 | 0.0001232 |  | Tolerated  (0.47) | Benign  (0.016) |
| 19 | 55327960 | KIR3DL1 | NM_013289.2 | c.5C>T | p.Ser2Leu | rs605219 | 0.0001192 |  | Tolerated  (0.13) | Benign  (0.017) |
| 19 | 55363704 | KIR3DL2 | NM_006737.3 | c.322G>A | p.Ala108Thr | - | 0 |  | Tolerated  (0.08) | Benign  (0.032) |
| 20 | 39831176 | ZHX3 | NM_015035.3 | c.2381A>G | p.Tyr794Cys | - | 0 |  | Deleterious  (0) | Probably damaging  (0.999) |
| 20 | 49551780 | DPM1 | NM_003859.1 | c.679-8dupT | - | rs11483542 | 0.0001917 |  | n.a. | n.a. |
| 22 | 19968941 | ARVCF | NM_001670.2 | c.689C>G | p.Pro230Arg | - | 0.0001227 |  | Deleterious  (0.01) | Probably damaging  (0.998) |
| 22 | 50964249 | TYMP | NM_001257989.1 | c.1414T>C | p.Phe472Leu | - | 0 |  | Tolerated  (0.66) | Benign  (0.007) |

**Supplementary Table S5**. **Results of Amplicon-based deep sequencing to Evaluate the Germline Nature of Detected *PLCG2* Variants.** ^1^NCBI Reference Sequence NM_002661.3. ^2^Mean of data collected from three independent experiments.

| **Family** | **Individual** | **Gene** | **Exon** | **cDNA alteration^1^** | **Predicted amino acid alteration** | **Amplicon-based deep sequencing** | |
| --- | --- | --- | --- | --- | --- | --- | --- |
|  |  |  |  |  |  | **Mutated allele frequency (%)^2^** | **Coverage^2^** |
| Family 1 | Patient | *PLCG2* | 24 | c.2533_2544del TTAGGGTCTCTT | p.(Leu845_Leu848del) | 51.8 | 3832x |
|  | Father | *PLCG2* | 24 | c.2533_2544del TTAGGGTCTCTT | p.(Leu845_Leu848del) | 0.0 | 1739x |
|  | Mother | *PLCG2* | 24 | c.2533_2544del TTAGGGTCTCTT | p.(Leu845_Leu848del) | 0.0 | 2291x |
| Family 2 | Patient | *PLCG2* | 20 | c.2122G>C | p.(Ala708Pro) | 51.2 | 2636x |
|  | Father | *PLCG2* | 20 | c.2122G>C | p.(Ala708Pro) | 0.1 | 6227x |
|  | Mother | *PLCG2* | 20 | c.2122G>C | p.(Ala708Pro) | 0.1 | 4025x |

**LEGENDS OF SUPPLEMENTARY FIGURES**

**Supplementary Figure S1.** Laboratory monitoring of two patients during the course of their disease. Gray areas represent the age-matched normal reference range for each parameter.

**Supplementary Figure S2.** Flow cytometric analysis of B cell differentiation stages in the bone marrow from patient 1.

**Supplementary Figure S3**. **Results of Ca^2+^ flux assay in Patient 2.** **Panel A**. Distribution of CD19^+^ and CD3^+^ lymphocyte subpopulations in peripheral blood mononuclear cells (PBMCs). **Panels B and C**. Intracellular Ca^2+^ content in CD3^-^ CD19^+^ cells before and after IgM crosslinking (panel B) and ionomycin (panel C) stimulation. Black arrows indicate concrete time of cells stimulation. Showed results are representative of one out of the two independent performed experiments.
