# Supplementary figures and images for: Severe Autoinflammatory Manifestations and Antibody Deficiency Due to Novel Hypermorphic PLCG2 Mutations
Source: J Clin Immunol. 2020 Jul 15;40(7):987–1000. doi: 10.1007/s10875-020-00794-7 (PMC7505877; doi:10.1007/s10875-020-00794-7)

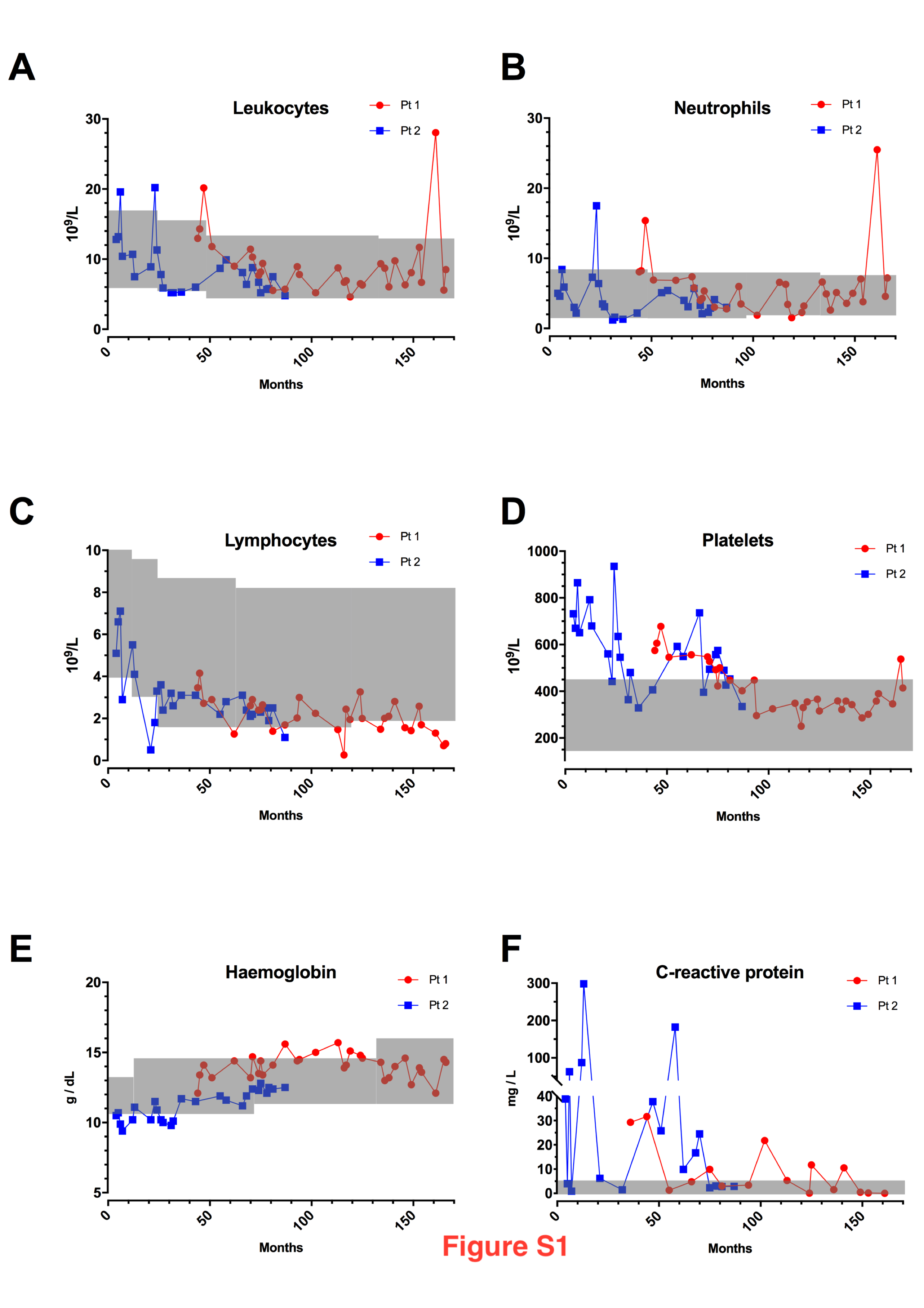

Supplement: Supplementary file 2 — (PNG 873 kb) [file 10875_2020_794_Fig5_ESM.png]
